# Study on the Antipyretic and Anti-inflammatory Mechanism of Shuanghuanglian Oral Liquid Based on Gut Microbiota-Host Metabolism

**DOI:** 10.3389/fphar.2022.843877

**Published:** 2022-06-28

**Authors:** Yan Gao, Lu Liu, Chen Li, Yu-Ting Liang, Jing Lv, Long-Fei Yang, Bo-Nian Zhao

**Affiliations:** Shandong University of Traditional Chinese Medicine, Jinan, China

**Keywords:** Shuanghuanglian oral liquid, metabolomics, gut microbiota, antipyretic, antiinflammatory

## Abstract

Nowadays, there has been increased awareness that the therapeutic effects of natural medicines on inflammatory diseases may be achieved by regulating the gut microbiota. Shuanghuanglian oral liquid (SHL), the traditional Chinese medicine preparation, has been shown to be effective in clearing heat-toxin, which is widely used in the clinical treatment of respiratory tract infection, mild pneumonia, and common cold with the wind-heat syndrome. Yet the role of gut microbiota in the antipyretic and anti-inflammatory effects is unclear. In this study, a new strategy of the 16S rRNA gene sequencing and serum metabolomics that aims to explore the role of SHL in a rat model of the systemic inflammatory response induced by lipopolysaccharide would be a major advancement. Our results showed that the gut microbiota structure was restored in rats with inflammation after oral administration of SHL, thereby reducing inflammation. Specifically, SHL increased the relative abundance of *Bacteroides* and *Faecalibacterium* and decreased the abundance of *Bifidobacterium*, *Olsenella*, *Aerococcus*, *Enterococcus*, and *Clostridium* in the rat model of inflammatory disease. Serum metabolomic profile obtained by the orbitrap-based high-resolution mass spectrometry revealed significant differences in the levels of 39 endogenous metabolites in the inflammatory model groups, eight metabolites of which almost returned to normal levels after SHL treatment. Correlation analysis between metabolite, gut microbiota, and inflammatory factors showed that the antipyretic and anti-inflammatory effects of SHL were related to the recovery of the abnormal levels of the endogenous metabolites (N-acetylserotonin and 1-methylxanthine) in the tryptophan metabolism and caffeine metabolism pathway. Taken together, these findings suggest that the structural changes in the gut microbiota are closely related to host metabolism. The regulation of gut microbiota structure and function is of great significance for exploring the potential mechanism in the treatment of lipopolysaccharide-induced inflammatory diseases with SHL.

## Introduction

Fever can be defined as a physiological stress response characterized by a higher body temperature than the upper limit of normal human temperature. This increase in tightly-regulated body temperature often exerts several adverse effects on clinical outcomes of various illnesses such as metabolic disorders and nerve damage (Bligh et al., 1973; Polderman, 2008; Onisor et al., 2019). As inflammation is one of the common causes of fever, inflammatory diseases are often accompanied by elevated body temperature. A myriad of research has highlighted the association of inflammatory diseases to gut microbiota (Wang et al., 2020). For instance, lipopolysaccharide (LPS) is a key endotoxin of gram-negative bacteria, and its movement to the systemic circulation is restricted by the intestinal barrier (Pickard et al., 2017). However, the leakage of LPS to systemic circulation due to dysregulated permeability of the gut triggers the cascade of inflammatory pathways and subsequently results in the activation and production of inflammatory cytokines, including interleukin 6 (IL-6), tumor necrosis factor *α* (TNF-α), and interferon *γ* (IFN- γ) (Do et al., 2016). Li et al. found that the gut microbiota of the rats having inflammation was enriched with four types of bacteria: *Streptococcus luteciae*, *Lactobacillus hamster*, *Bacteroides uniformis*, and *Bacteroides ovatus* (Liang et al., 2014). A significant change in the composition and diversity of commensal flora of the gut has been reported in relation to the treatment with non-steroidal anti-inflammatory drugs (NSAIDs) (Liang et al., 2015).

The side effects of many commercially available antipyretic and anti-inflammatory drugs on gastrointestinal, cardiovascular, and nervous systems (Bozimowski, 2015) can create difficulty for physicians and researchers in weighing the pros and cons of these treatments. In recent years, physicians and patients have recognized natural and traditional Chinese medicines owing to their antipyretic and anti-inflammatory potential with minimal collateral damage. The Chinese herbal preparation Shuanghuanglian oral liquid (SHL) is widely used in the clinical treatment of inflammatory diseases, such as pneumonia and acute tonsillitis, and it is composed of three botanical drugs, namely *Lonicera japonica* Thunb. (Caprifoliaceae; Flos Lonicerae japonicae), *Scutellaria baicalensis* Georgi (Lamiaceae; Radix Scutellariae) and *Forsythia suspensa* (Thunb.) Vahl. (Oleaceae; Fructus Forsythiae) (The State Commission of Chinese Pharmacopoeia, 2020). These botanical drugs have three active ingredients, namely chlorogenic acid, baicalin, and forsythin, which were formally recorded as quality control standards in the 2015 Pharmacopoeia (Commission, 2015). Although these active ingredients have low oral bioavailability, these chemical entities are easily metabolized by the gut microbiota and mostly decompose to metabolites of lower molecular weight and polarity and consequently yield better bioavailability (Feng et al., 2019). Studies have confirmed that human fecal microflora could transform baicalin to baicalein and oroxylin A, which exhibit a more potent anti-scratching behavioral effect than their parent molecule in histamine-treated mice (Jung et al., 2012; Cheng et al., 2021). Moreover, the bioavailability of herbal compounds can be further improved by their possible effect on the production of microbial metabolites (Zhang et al., 2020; Liu et al., 2022). Therefore, gut microbiota combined with metabolomics is widely used to study the mechanism of Chinese medicine (Feng et al., 2021).

A high-throughput metagenomics-based determination method for microorganisms, viz, 16S rRNA gene sequencing technology, is often used for structural identification and prediction of the metabolic function of gut microbiota (Shang et al., 2018). Due to high sensitivity and ultra-high performance liquid chromatography–mass spectrometry (UHPLC-MS)-based non-targeted metabolomics, 16S rRNA gene sequencing technology can reveal potential clinical biomarkers and describe possible pathogenesis of various ailments, including cardiovascular, neurological, and oncological diseases, through comprehensive monitoring of metabolites (Jian and Weng, 2017). Thus, this technology could be exploited to study the metabolic pathways in disease- or drug-affected commensal flora of the gut and to eventually find the relevant biomarkers and drug treatment targets. The mechanism of SHL effects on host serum metabolism through gut microbiota remains to be elucidated. Furthermore, exploration of the preventive and therapeutic impact of traditional Chinese medicine on fever and LPS-induced inflammation can provide a new perspective for the clinical treatment of systemic inflammatory responses.

The present study aimed to explore the effects of SHL on the gut microbiota and biomarkers of LPS-induced inflammation in rats and to examine the potential connection between serum metabolites and gut microbiota. Since the presence of drug-derived components in the serum may serve as a good indicator for the *in vivo* efficacy of Chinese medicine, the current study employed UHPLC-MS non-targeted metabolomics to analyze and compare the inflammation in control and SHL-treated groups, whereas the 16S rRNA sequencing technology was used to study the possible connection between the commensal flora of the gut and serum metabolites by exploring the changes of gut microbiota before and after administration of SHL. These evaluations can provide a more comprehensive and detailed understanding of the mechanism of SHL in alleviating LPS-induced fever and inflammation through regulating the host gut microbiota.

## Materials and Methods

### Chemicals and Drugs

SHL samples were purchased from Harbin Pharmaceutical Group Sanjing Pharmaceutical Co., Ltd. (China, batch number: 20081311). LPS was purchased from Shanghai Yuanye Bio-Technology Co., Ltd. (China, batch number: M11GS141189). Aspirin was purchased from Cisen Pharmaceutical Co., Ltd. (China, batch number: 2005112512). Cholic acid-2,2,3,4,4,-d5 (CAS: 53007-09-3; purity ≥98%) and succinic acid-2,2,3,3,-d4 (CAS: 14993-42-6; purity ≥99%) were purchased from Sigma Company (United States). DL-tryptophan-2,3,3-d3 (CAS: 340257-61-6; purity ≥98%) and DL-methionine-3,3,4,4,-d4 (CAS: 93709-61-6; purity ≥99%) were purchased from CDN Company (China). L-phenylalanine (Ring-D5) (CAS: 63-91-2; purity ≥98%) and choline chloride (Trimethyl-D9) (purity ≥98%) were purchased from CIL Company (United States). Agarose (batch number: 75510-019) was purchased from Invitrogen (United States, batch number: DL15000). The marker was purchased from Takara (Japan). TAE (batch number: AM9870) was purchased from Invitrogen (United States). Quant-iT PicoGreen dsDNA assay kit (batch number: P7589) was purchased from Invitrogen (United States). Q5® High-Fidelity DNA polymerase (lot number: M0491L) was purchased from Beijing NBE (China). Agilent High Sensitivity DNA kit and MiSeq Reagent Kit V3 (600 cycles, Illumina, United States) were used. Methanol and acetonitrile (purity ≥99.0%) were purchased from Thermo (United States). Ammonium formate (purity ≥99.0%) was purchased from Sigma (United States). Formic acid was of LC-MS grade, and other reagents were of analytical purity. All rat-specific enzyme-linked immunoassay (ELISA) kits were purchased from Multi Science (Lianke) Biotech, Co., Ltd., Hangzhou, China.

### Pretreatment Procedure for Drug

According to the original composition and preparation method of Shuang-Huang-Lian oral liquid recorded in the Chinese Pharmacopeia (The State Commission of Chinese Pharmacopoeia, 2020), it was prepared by the following procedure. 375 g of *Scutellaria baicalensis* Georgi (Lamiaceae; Radix Scutellariae), 375 g of *Lonicera japonica* Thunb. (Caprifoliaceae; Flos Lonicerae japonicae), and 750 g of *Forsythia suspensa* (Thunb.) Vahl. (Oleaceae; Fructus Forsythiae) were decocted by boiling for 2 h once and then boiling for 1 h twice, concentrated, extracted with ethanol, adjusted PH value with HCl and NaOH, distilled to eliminate solvent, and the residue was dissolved and diluted with water to 1000 ml in volume. The extracted solution was filtered and made to a concentration of 1.5 g raw material per milliliter. In this study, the concentration of SHL preparation (batch number: 20081311) was 1.5 g mL^−1^. LPS and aspirin were weighed accurately and dissolved in normal saline into a 50 ml volumetric flask separately to obtain 100 μg mL^−1^ LPS stock solution and 10 mg mL^−1^ aspirin solution.

### Animals Treatments and Sample Collection

Healthy adult male Wistar rats (weight: 220 ± 20 g, purchased from Jinan Pengyue Experimental Animal Breeding Co., Ltd., Shandong, China) were adaptively fed for 3 days in standard laboratory conditions at Shandong University of Traditional Chinese Medicine. After passing the quarantine, they were randomly divided into six groups, namely the normal control group (CG), the model group (MG), positive drug group (PG), SHL low (LG), medium-dose group (ZG), and high-dose group (HG), with eight rats each group. For three consecutive days prior to the LPS intervention, an SHL oral dose of 6.3, 12.6, and 25.2 ml kg^−1^ was administrated once a day to the LG, ZG, and HG groups, respectively. After fasting for 24 h, the LG, ZG, and HG groups were orally administered with SHL again. The CG and PG were injected with an equal volume of normal saline and aspirin (100 mg kg^−1^) after 0.5 h. Then, LPS solution (100 μg kg^−1^) was intraperitoneally injected into each group except CG. The body temperature level of rats in each group was detected every 30 min. Blood samples were collected from the inner canthus of the rats under ether anesthesia at different time points. Then, 300 mg of intestinal excreta were collected in the sterile tube and stored in liquid nitrogen immediately. Serum samples were placed at room temperature for 30 min and then centrifuged at 3000 rpm for 10 min. All samples were stored at −80°C before analysis.

### 16S rRNA Microbial Community Analysis

Differences in gut microbiota among the CG, MG, and the optimal concentration of SHL administration group were assessed by collecting intestinal excreta samples from four randomly selected rats in each group for 16S rRNA gene analysis on the Illumina MiSeq platform. Total DNA was obtained using a DNA gel extraction kit. Furthermore, the purity and concentration of total DNA employed in this experiment were analyzed using a spectrophotometer, and DNA integrity was detected by 1.2% agarose gel electrophoresis. The V3–V4 region of the 16S rRNA gene was amplified by polymerase chain reaction (PCR) with the following primers: 338F (5′-ACT​CCT​ACG​GGA​GGC​AGC​A-3′) and 806R (5′-GGACTACHVGGGTWTCTAAT-3′). PCR assay was performed at 95°C for 3 min, followed by 27 cycles of 95°C for 30 s, 55°C for 30 s, and 72°C for 45 s, with the last step at 72°C for 10 min. The amplified PCR products were excised from 2% agarose gels and purified using the AxyPrep DNA Kit. The concentration of libraries was determined using a Qubit 2.0 fluorometer (Invitrogen) with the Quant-it PicoGreen dsDNA Assay Kit (Thermo Scientific).

According to the overlapping relationship between the paired-end reads, the sequence data achieved by HiSeq sequencing were merged into a sequence of tags, and the quality of reads was controlled and filtered. After discriminating samples, community bar charts were analyzed at phylum and genus levels. β-diversity was further studied by the Bray–Curtis distance algorithm and visualized by principal coordinate analysis (PCoA) (Bray and Curtis, 1957). Phylogenetic investigation of communities by reconstruction of unobserved states (PICRUSt) was used to predict the abundance of gene categories (COGs) and microbiota-derived pathways (Gavin et al., 2019). The description and functional information of each COG were parsed from the eggNOG database to obtain a functional abundance profile.

### Serum Metabolomics Analysis

Internal standards solution includes succinic acid-2,2,3,3,-d4 (30 ppm final concentration), cholic acid-2,2,3,4,4-d5 (45 μM final concentration), L-phenylalanine-d5 (15 μM final concentration), DL-methionine-3,3,4,4-d4 (30 μM final concentration), DL-tryptophan-d3 (15 μM final concentration), and choline chloride-d9 (15 μM final concentration). Serum samples (100 μL) were added to methanol solution (400 μL) and the mixed solution of internal standards (100 μL). Then, the mixture was vortexed for 1 min with centrifugation for 10 min at 12,000 rpm, 4°C. The supernatant (500 μL) was dried under a vacuum concentrator. It was re-dissolved in 80% methanol solution (150 μL), thoroughly mixed and centrifuged (12000 rpm, 10 min, 4°C). Quality control (QC) samples were prepared to balance the chromatography–mass spectrometry for evaluating the stability of the system during the entire experiment. 20 µL of each extract supernatant was mixed to form a single QC sample for ultra-high-field X orbitrap mass spectrometry analysis (Sangster et al., 2006; Zelena et al., 2009; Dunn et al., 2011).

### Ultra-High-Field X Orbitrap Mass Spectrometry Conditions

Serum samples were analyzed on the Waters Acquity UPLC System (Milford, MA, United States). The Acquity UPLC HSS T3 C18 column (2.1 mm × 150 mm, 1.8 μm; Waters, Milford, MA, United States) was selected. In positive mode, the mobile phases were as follows: A1: 0.1% aqueous formic acid solution and B1: 0.1% formic acid acetonitrile solution. The mobile phases in negative ion mode were A2: 5 mM ammonium formate water solution and B2: acetonitrile solution. The injection volume was 2 μL, and the flow rate was 250 μL/min. The column temperature was set to 40°C. The gradient elution procedure was as follows: (0–1 min), 2% B1/B2; (1–9 min), 2–50% B1/B2; (9–12 min), 50–98% B1/B2; (12–13.5 min), 98% B1/B2; (13.5–14 min), 98–2% B1/B2; (14–20 min), 2% B1-positive mode; (14–17 min), and 2% B2-negative ion mode. Ultra-high-field X Orbitrap mass spectrometer (Thermo, United States) and electrospray ionization (ESI) were used for mass spectrometry detection with positive and negative ion modes. The spray voltages of positive ions and negative ions were 3.50 and 2.50 kV, respectively. Other parameters were consistent, and the setting was as follows: sheath gas, 30 arb; auxiliary gas, 10 arb; capillary temperature, 325°C; resolution, 60000; and scan range, 81–1000 m/z. The secondary cracking was performed with HCD at a collision voltage of 30 eV, and unnecessary MS/MS information was removed by using the dynamic exclusion method.

### Mathematical Statistical Analysis

ProteoWizard software was used to convert the high-resolution mass spectrometry data into mzXML format and R software (v3.3.2, XCMS kernel) was used for retention time correction, peak identification, peak extraction, and peak alignment. The metabolites were identified based on accurate mass (mass measurement accuracy within 10 ppm). After that, metabolites were obtained according to the fragmentation pattern of MS/MS and confirmed by Metlin (http://metlin.scripps.edu), MoNA (https://mona.fiehnlab.ucdavis.edu/), and a self-built standard database. The dataset was manually corrected to remove system contaminants and uninformative data by comparing with the blank sample and then normalized to sample weight before various statistical analyses were conducted. Differential metabolite analysis consisted of UPLC-MS data preprocessing and multivariate analysis such as principal component analysis (PCA) and orthogonal partial least squares discrimination analysis (OPLS-DA). PCA was used to get an overview of sample distribution and possible outliers. OPLS-DA was carried out to identify significant metabolites. The quality of the OPLS-DA model was assessed by a 7-fold cross-validation method. R^2^Y and Q^2^ values were presented to show the ability of the model to explain y matrix information and the predictability of the model, which was calculated by SIMCA-P^+^ software (v14.1, Umetrics AB, Umea, Sweden). In order to further verify the fitting degree of the model, permutation tests were performed many times (*n* = 200) randomly to get the corresponding random Q^2^ values. According to the results of OPLS-DA, variable importance projection (VIP) > 1 and *p*-value < 0.05 were considered potential chemical markers. Characteristic metabolites were further identified by the combination of secondary fragment ions and parent ions.

### Determination of Serum Biochemical Indicators

The serum concentrations of pyrogenic cytokines such as IL-6, interleukin-1β (IL-1β), and TNF-α were determined using rat-specific ELISA. In this study, all samples were tested in parallel with the secondary hole. For non-parametric distribution, the Kruskal–Wallis test complemented with Dunn’s tests was performed to determine statistical significance (*indicates *p*-value < 0.05).

## Results

### Effect of Shuanghuanglian Oral Liquid Treatment on Physiological and Biochemical Indicators

**FIGURE 1:**
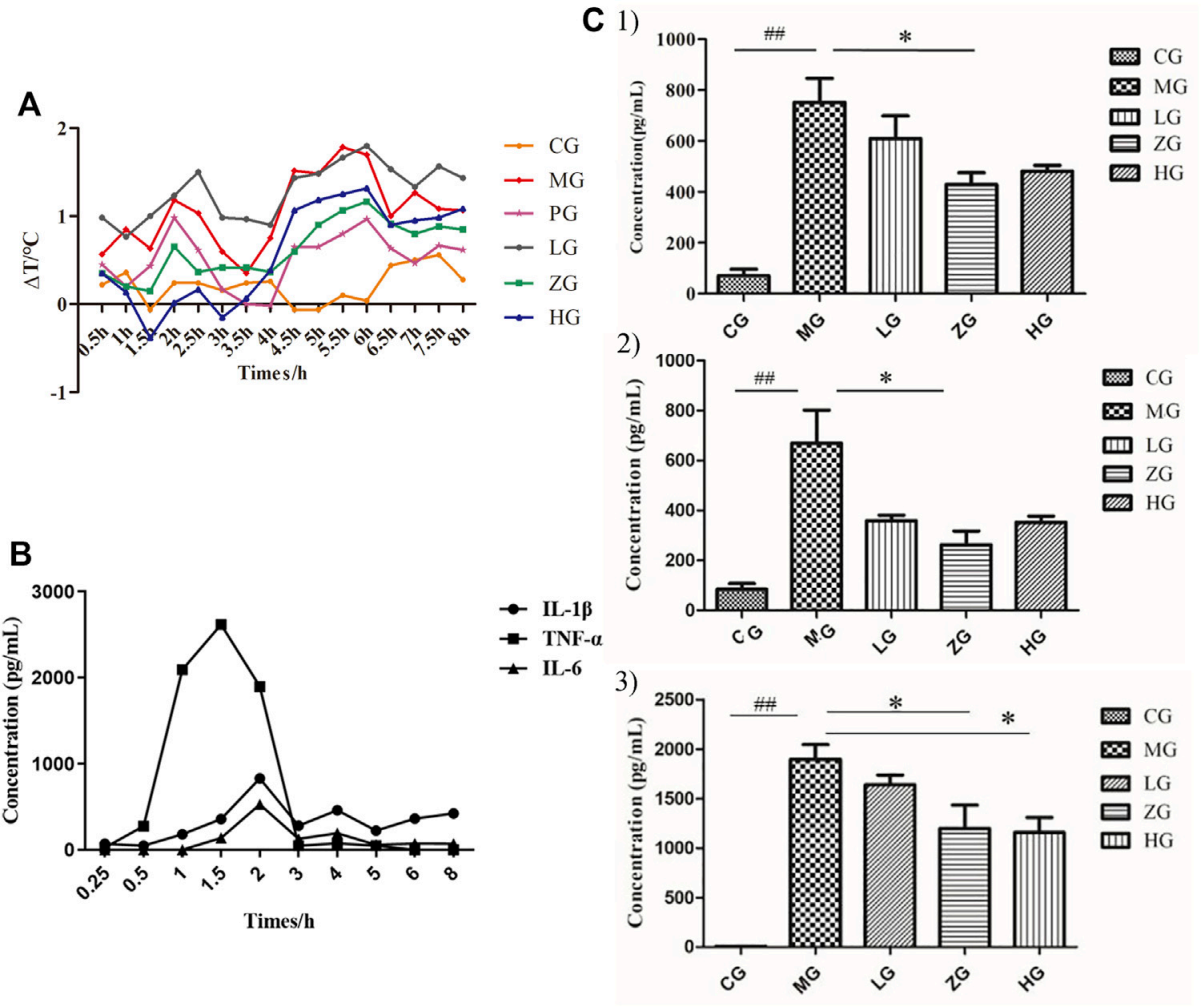
Regulation of physiological and biochemical indicators levels induced by LPS and its reduction by SHL treatment. **(A)** The trends of temperature difference to time (ΔT) of rats in each group (*n* = 8); **(B)** Changes in the level of pro-inflammatory cytokines (IL-1β, IL-6, and TNF-α) of serum samples collected at different time points in model groups; **(C)** The levels of pro-inflammatory cytokines of serum samples at 2 h-point blood sampling. (1: IL-1β; 2: IL-6; and 3: TNF-α). Data were expressed as mean ± SD (*p < 0.05 model groups compared with the SHL treatment groups, ^*##*^ *p* < 0.01 compared with the control groups, CG, normal control group, MG, model group, LG, SHL low-dose group, ZG, SHL medium-dose group; HG, SHL high-dose group).

To investigate the antifebrile effects of SHL, we recorded the rectal temperatures of rats continuously for 8 h after LPS infusion (Figure 1A, Supplementary Table S1). The results showed that the rectal temperature significantly increased in a three-phase thermal curve (peaks at ∼1, 2, and 5.5 h postinjection) after injecting LPS. In addition, ZG and HG could markedly decrease the temperature at almost all the checking points, indicating the antifebrile effect of SHL. However, HG exhibited lower body temperature than CG in the prefebrile phase. To study the optimal time point for blood sampling, concentrations of pro-inflammatory cytokines in CG, MG, and ZG at different blood collection time points were recorded. As shown in Figure 1B, TNF-α was detected within 30 min after LPS invasion and reached the maximum value earlier than the other two cytokines, which was consistent with other studies (Wang et al., 2019). All three pro-inflammatory cytokines reached high response values at 2nd h, and the contents of the three cytokines decreased significantly after SHL treatment (Supplementary Figure S1). That was why the optimal time point for 2 h-point blood sampling was selected for subsequent metabolomic analysis. As shown in Figure 1C, the non-parametric Mann–Whitney U test of the comparison between different groups found that compared with the normal control group, the levels of IL-1β (^*##*^ *p* < 0.01), IL-6 (^*##*^ *p* < 0.01), and TNF-α (^*##*^ *p* < 0.01) pro-inflammatory cytokines at 2 h time point were remarkably increased in rats with inflammation. And the levels of three pro-inflammatory cytokines decreased significantly after SHL treatment with medium doses (*p < 0.05). Thus, the optimal dose group (ZG) was selected for further serum metabonomics and 16S rRNA microbial community analysis.

### Serum Metabolic Profile of the Rat Model of Inflammation and Identification of Potential Metabolite Biomarkers

Serum metabolomics can be used to find all the different metabolites among samples and the characteristic markers of biological systems by analyzing the composition and content changes of small-molecule chemicals (metabolites) in samples (Tuttolomondo et al., 2008; Zhang et al., 2012). In the relative quantitative analysis of metabolomics, the chemical components of CG, MG, and ZG were detected by ultra-high-field X Orbitrap mass spectrometer. Typical base peak intensity chromatograms of serum samples in positive and negative ion modes are illustrated in Supplementary Figure S2. Batch normalization was used to screen real biological signals, resulting in a data matrix containing 24 samples and 20,514 variables in positive ion mode. The data matrix containing 24 samples and 12,377 variables was obtained in negative ion mode. After further analysis of the filtered data, a total of 58 metabolites in negative ion and 127 metabolites in positive ion were identified, including amino acids, lipids, carbohydrates, nucleotides, xenobiotics, cofactors, and vitamin compounds.

**FIGURE 2:**
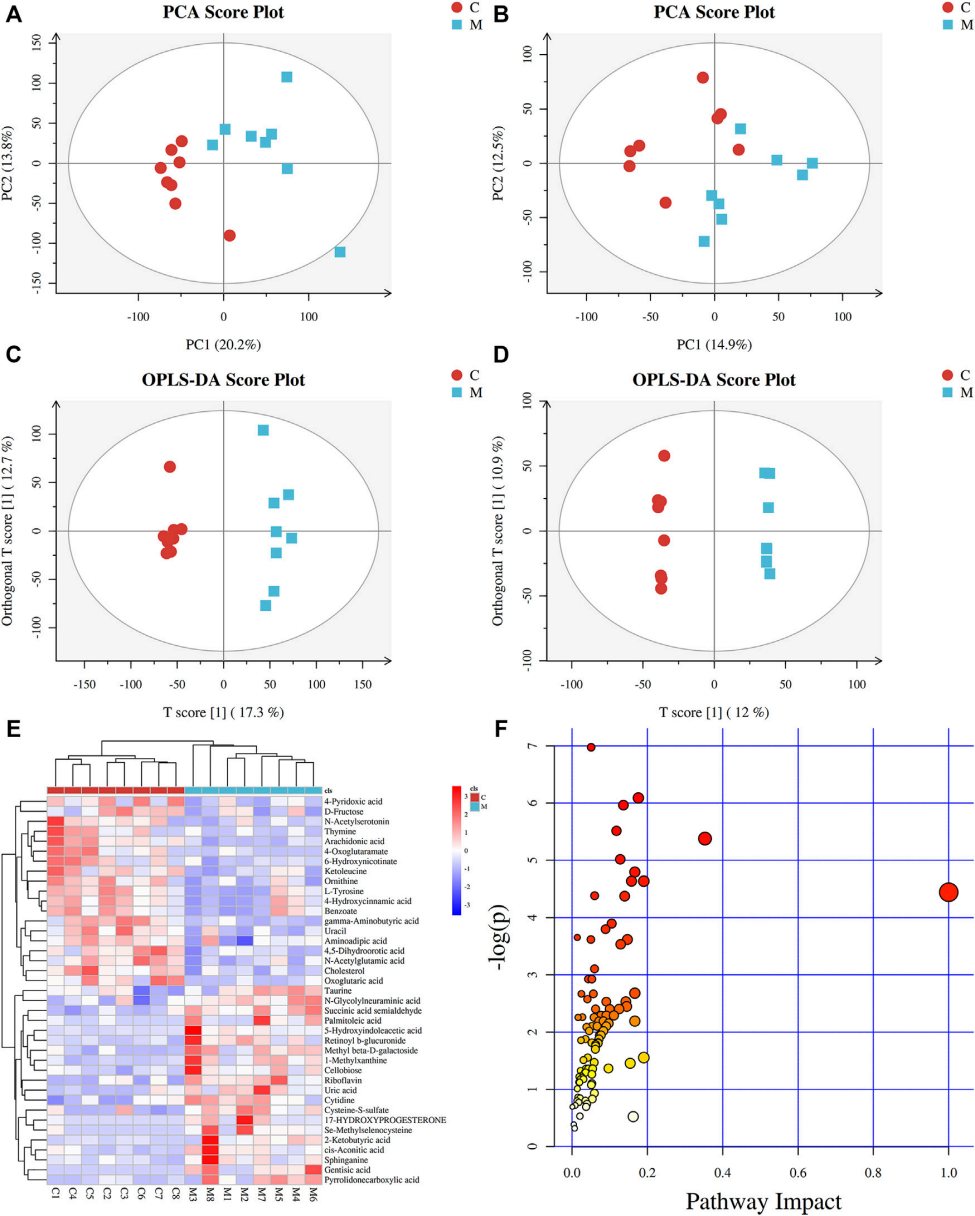
Metabolic profiles of serum samples in rats with inflammation induced by LPS. **(A)** PCA score plot in positive mode; **(B)** PCA score plot in negative mode; **(C)** OPLS-DA score plot in positive mode (R^2^Y = 0.980 and Q^2^Y = 0.803); **(D)** OPLS-DA score plot in negative mode (R^2^Y = 0.999 and Q^2^Y = 0.680); **(E)** Clustering heatmaps of differential metabolites between the normal control (CG) and model (MG) groups. Red represents high adjacency (positive correlation), and blue represents low adjacency (negative correlation); **(F)** Metabolic pathway analysis of differential metabolites between the normal control (CG) and model (MG) groups.

In order to identify the metabolic characteristics of serum samples from each group, multivariate statistical methods were used to analyze the normalized metabolic data. Principal component analysis (PCA) and orthogonal projections to latent structures discriminant analysis (OPLS-DA) were used to explore the differential variables. PCA score plot showed that the serum datasets of different groups could be separated in positive ion mode (Supplementary Figure S3A) but not significantly distinguished in negative ion mode (Supplementary Figure S3B). No abnormal samples were found according to the DModX plot, after which OPLS-DA analysis was performed. The scores plots of PCA and OPLS-DA in positive and negative ion modes between CG and MG are shown in Figure 2. According to the PCA score plots (Figures 2A and B), there was an incomplete separation between CG and MG, and one outlier was found. Then, the OPLS-DA model was often used to analyze standardized data and identify marker compounds because of its ability to minimize the differences between groups. According to the OPLS-DA score plots (Figures 2C and D), the samples could be divided into two categories clearly, and the model established had no risk of overfitting in the 200 permutation tests (Supplementary Figures S4A and B). Furthermore, OPLS-DA combined with VIP value (threshold >1) and *p*-value (threshold <0.05) was often used to determine the potential chemical markers. Comparing the inflammatory MG with the CG, 31 metabolites showed significantly different levels in the positive ion mode and 8 in the negative ion mode (Table 1). Differentially expressed biomarkers mainly included lipid, carbohydrate, amino acid, nucleotide, pyrimidine, neurotransmitter, and vitamin compound. Changes in identified serum biomarkers associated with LPS-induced inflammatory hyperthermia were described in heatmaps (Figures 2G and H). Topological metabolic pathway analysis was performed for all 39 potential biomarkers using KEGG and MetPA databases. The metabolic network associated with the inflammatory hyperthermia model is shown in Table 1. For serum samples in rats with LPS-induced inflammation, the metabolic pathways were determined as D-glutamine and D-glutamate metabolism, riboflavin metabolism, cholesterol and arachidonic acid metabolism, arginine and proline metabolism, taurine and hypotaurine metabolism, butanoate metabolism, pyrimidine metabolism, alanine, aspartate, and glutamate metabolism, and tryptophan metabolism.

**TABLE 1:** Differential metabolite identification results between normal control group and model group.

No.	Metabolites	Mass observed (m/z)	t_R_ (s)	Molecular ion	VIP	C vs. M	*p-*value
1	Arachidonic acid	303.2329	832.067	[M−H]^−^	2.26	↓***	0.0009
2	L-Tyrosine	180.0659	182.606	[M−H]^−^	2.03	↓**	0.0074
3	Ketoleucine	129.0543	336.209	[M−H]^−^	1.94	↓**	0.0054
4	gamma-aminobutyric acid	104.1069	86.6878	[M + H]^+^	1.92	↓**	0.0019
5	Ornithine	131.0818	93.2464	[M−H]^−^	1.91	↓*	0.0136
6	Succinic acid semialdehyde	103.0392	35.4215	[M + H]^+^	1.89	↑**	0.0028
7	N-glycolylneuraminic acid	324.0935	81.1393	[M−H]^−^	1.86	↑*	0.0181
8	Cysteine-S-sulfate	199.9684	82.1101	[M−H]^-^	1.79	↑*	0.0239
9	Cholesterol	387.1899	791.37	[M + H]^+^	1.77	↓**	0.0019
10	6-hydroxynicotinate	138.0196	602.946	[M−H]^−^	1.77	↓*	0.0313
11	Methyl beta-D-galactoside	176.9716	38.0247	[M + H−H_2_O]^+^	1.73	↑**	0.0054
12	N-acetylglutamic acid	190.0708	197.119	[M + H]^+^	1.73	↓**	0.0054
13	4,5-dihydroorotic acid	158.9607	189.9105	[M + H]^+^	1.72	↓**	0.0054
14	Riboflavin	377.1442	441.7005	[M + H]^+^	1.7	↑**	0.0074
15	1-methylxanthine	166.0494	325.166	[M]^+^	1.65	↑***	0.0007
16	Oxoglutaric acid	147.0285	143.749	[M + H]^+^	1.65	↓**	0.0054
17	Cytidine	243.9402	78.4785	[M + H]^+^	1.63	↑**	0.0074
18	Uracil	182.9825	363.835	[M]^+^	1.62	↑*	0.0136
19	4-oxoglutaramate	145.0478	360.7435	[M]^+^	1.58	↓**	0.0054
20	Gentisic acid	154.9898	646.6	[M + H]^+^	1.58	↑**	0.0046
21	Pyrrolidonecarboxylic acid	130.0487	508.522	[M + H]^+^	1.58	↑**	0.0022
22	D-fructose	179.054	205.52	[M−H]^−^	1.53	↓*	0.0406
23	Uric acid	169.0347	100.036	[M + H]^+^	1.5	↑**	0.0074
24	Retinoyl b-glucuronide	476.2754	798.921	[M]^+^	1.48	↑*	0.0181
25	4-hydroxycinnamic acid	165.0542	220.229	[M + H]^+^	1.47	↓**	0.0037
26	N-acetylserotonin	219.1122	443.8	[M + H]^+^	1.45	↓**	0.0027
27	4-pyridoxic acid	182.9844	231.234	[M]^+^	1.43	↓*	0.0313
28	Cellobiose	343.1213	89.1744	[M + H]^+^	1.35	↑*	0.0101
29	Taurine	125.9862	104.32	[M + H]^+^	1.34	↑*	0.0313
30	Thymine	127.05	36.72315	[M + H]^+^	1.34	↓*	0.0406
31	Se-methylselenocysteine	113.0342	143.063	[M + H]^+^	1.33	↓*	0.0136
32	Aminoadipic acid	162.076	97.3018	[M + H]^+^	1.32	↓*	0.0313
33	cis-aconitic acid	175.0234	164.744	[M + H]^+^	1.31	↑*	0.0101
34	2-ketobutyric acid	102.034	159.9945	[M]^+^	1.27	↑*	0.0136
35	Benzoate	123.0433	220.229	[M + H]^+^	1.27	↓*	0.0406
36	Palmitoleic acid	254.2479	814.4685	[M]^+^	1.23	↑*	0.0496
37	17-HYDROXYPROGESTERONE	331.2255	770.4725	[M + H]^+^	1.21	↑**	0.0071
38	5-hydroxyindoleacetic acid	192.0639	438.305	[M + H]^+^	1.19	↑**	0.0009
39	Sphinganine	302.3035	778.696	[M + H]^+^	1.17	↑*	0.0181

Note: Compared with the normal control group; *p < 0.05, **p < 0.01, and ***p < 0.001; “↓” indicate decrease in normal controls group compared with the model group; “↑” indicate increase in normal controls group compared with the model group.

### Effect of Shuanghuanglian Oral Liquid Treatment on Serum Metabolomics

**FIGURE 3:**
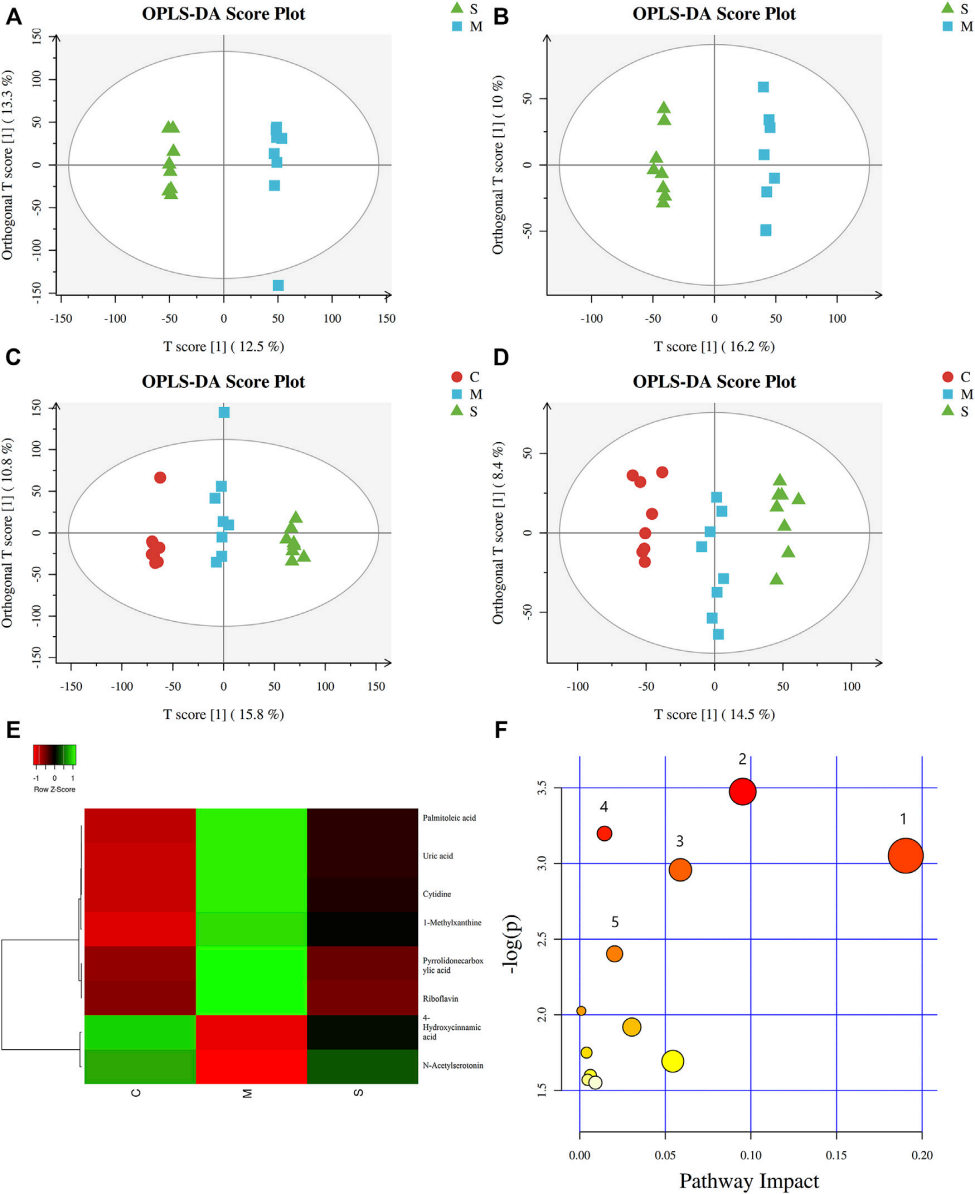
Metabolic profiles of serum samples in rats with SHL treatment in model groups. **(A)** OPLS-DA score plot in positive mode between model groups and SHL medium-dose groups (R^2^Y = 0.999 and Q^2^Y = 0.653); **(B)** OPLS-DA score plot in negative mode between model groups and SHL medium-dose groups (R^2^Y = 0.995 and Q^2^Y = 0.780); **(C)** OPLS-DA score plot in positive mode between normal control model, model groups and SHL medium-dose groups (R^2^Y = 0.995 and Q^2^Y = 0.888); **(D)** OPLS-DA score plot in negative mode between normal control model, model groups and SHL medium-dose groups (R^2^Y = 0.984 and Q^2^Y = 0.839); **(E)** Clustering heatmaps of differential metabolites between the normal control (CG), model (MG), and SHL medium-dose groups (ZG). Green represents high adjacency (positive correlation), and red represents low adjacency (negative correlation); **(F)** Metabolic pathway analysis of differential metabolites between the model groups (MG) and SHL medium-dose groups (ZG).

**FIGURE 4:**
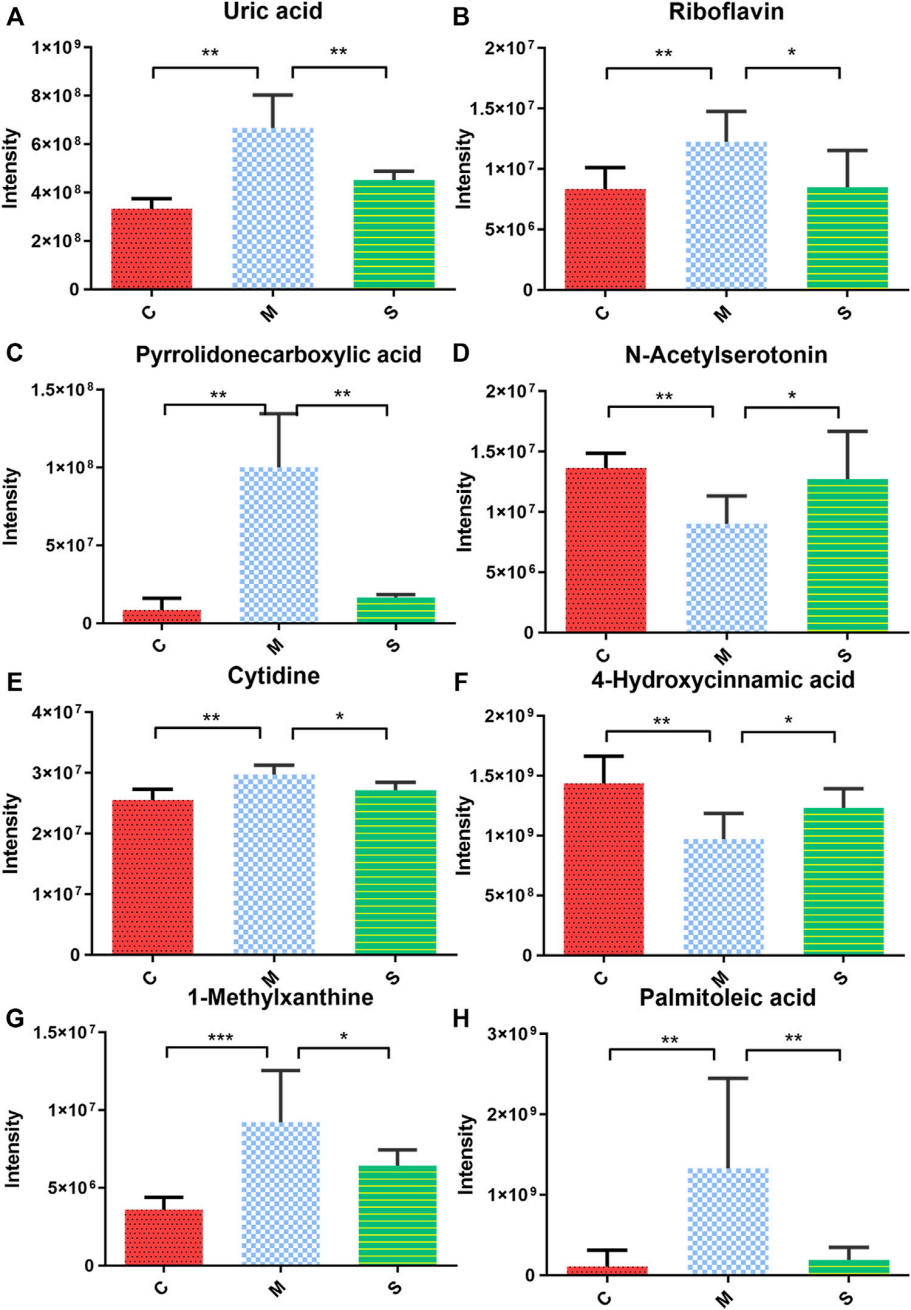
Expression of serum biomarkers in all groups, *p < 0.05, **p < 0.01 the model groups compared with the other groups; **(A)** Uric acid; **(B)** Riboflavin; **(C)** Pyrrolidonecarboxylic acid; **(D)** N-Acetylserotonin; **(E)** Cytidine; **(F)** 4-Hydroxycinnamic acid; **(G)** 1-Methylxanthine; and **(H)** Palmitoleic acid.

In order to explore the effect of SHL on the serum metabolomics of LPS-induced inflammation rats, OPLS-DA analysis was performed on data from all groups (CG, MG, and ZG) to obtain comprehensive metabolic characteristics. According to the OPLS-DA score charts of the three groups (Figures 3A–D), the ZG and the CG were significantly different from the MG on both sides of the Y-axis, indicating significant differences between the groups. In addition, in order to further verify the fitting degree of the model, permutation tests were performed many times (*n* = 200) randomly to get the corresponding random Q^2^ values. The results showed that the established OPLS-DA model was reliable and had good predictability (Supplementary Figures S4C and D). After treatment with SHL, the eight endogenous metabolites disordered in rats with inflammatory hyperthermia returned to almost normal levels (Figures 3E and F). As shown in Figure 4 and Table 2, the non-parametric Mann–Whitney U test of the comparison between the groups found that the eight different metabolites had a significant callback trend after the administration, such as uric acid, riboflavin, pyrrolidonecarboxylic acid, N-acetylserotonin, cytidine, 4-hydroxycinnamic acid, 1-methylxanthine, and palmitoleic acid. These potential biomarkers were related to riboflavin metabolism, D-glutamine and D-glutamate metabolism, caffeine metabolism, ABC transporters, and vitamin rare earths and absorption. They were respectively attributed to coenzyme factor and vitamin metabolism, amino acid metabolism, exogenous biodegradation and metabolism, nucleotide metabolism, and other upstream metabolic pathways, so it is speculated as potential target pathways for SHL to exert antipyretic and anti-inflammatory effects.

**TABLE 2:** Characterization of differential metabolites and metabolic pathways in SHL medium-dose group.

No.	Metabolites	Mass observed (m/z)	t_R_ (s)	Molecular ion	Formula	KEGG	Sub pathway	Super pathway
1	Uric acid^**##^	169.0347	100.04	[M + H]^+^	C_5_H_4_N_4_O_3_	C00366	Purine metabolism, Bile secretion	Nucleotide metabolism, Digestive system
2	Riboflavin^**#^	377.1442	441.70	[M + H]^+^	C_17_H_20_N_4_O_6_	C00255	ABC transporters, Vitamin digestion and absorption, Riboflavin metabolism	Membrane transport, Digestive system, Metabolism of cofactors and vitamins
3	Pyrrolidonecarboxylic acid^**##^	130.0487	508.52	[M + H]^+^	C_5_H_7_NO_3_	C02237	D-Glutamine and D-glutamate metabolism	Amino acid metabolism
4	N-Acetylserotonin^**#^	219.1122	443.80	[M + H]^+^	C_12_H_14_N_2_O_2_	C00978	Tryptophan metabolism	Amino acid metabolism
5	Cytidine^**#^	243.9402	78.48	[M + H]^+^	C_9_H_13_N_3_O_5_	C00475	ABC transporters, Pyrimidine metabolism	Membrane transport, Nucleotide
6	4-Hydroxycinnamic acid^**#^	165.0542	220.23	[M + H]^+^	C_9_H_8_O_3_	C00811	Ubiquinone and other terpenoid-quinone, Tyrosine metabolism	Metabolism of cofactors and vitamins, Amino acid metabolism
7	1-Methylxanthine^***#^	166.0494	325.17	[M]^+^	C_6_H_6_N_4_O_2_	C16358	Caffeine metabolism	Biosynthesis of other secondary metabolites
8	Palmitoleic acid^**##^	254.2479	814.47	[M]^+^	C_16_H_30_O_2_	C08362	Fatty acid biosynthesis	Lipid metabolism

Note: Comparison between model group and normal control group; *p < 0.05, **p < 0.01, and ***p < 0.001; Comparison between SHL medium-dose group and model group; ^#^ *p* < 0.05 and ^##^ *p* < 0.01.

### Diversity Analysis of Gut Microbiota in Fecal Samples

**FIGURE 5:**
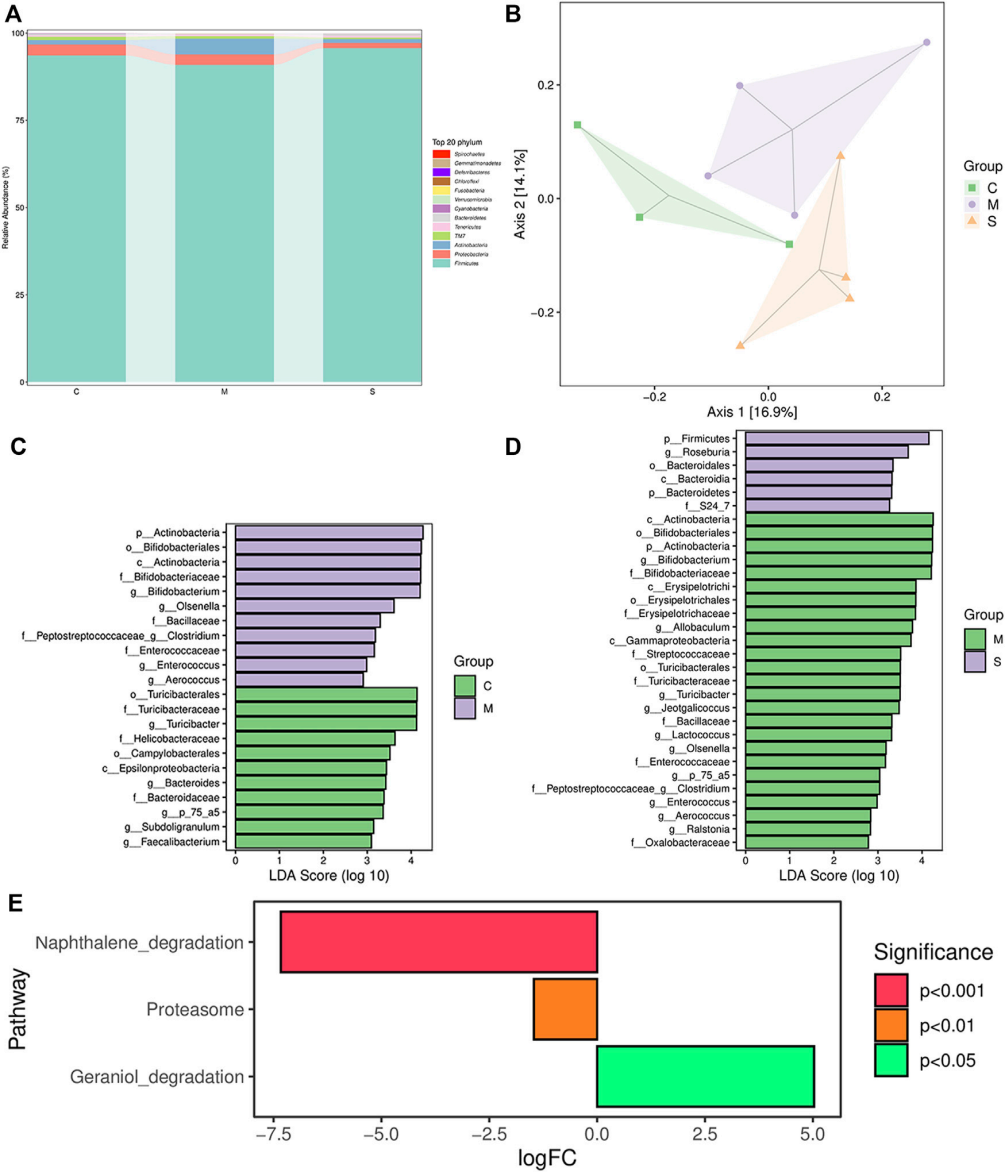
Results of 16S rRNA sequence analysis. **(A)** Overview of the gut microbial composition of the normal control groups, model groups, and SHL medium-dose groups at the phylum level. **(B)** PCoA of gut microbiota in the normal control groups and model groups. **(C)** LDA histogram of the normal control groups and inflammatory model groups. **(D)** LDA histogram of SHL medium-dose groups and inflammatory model rats. **(E)** Metagenomeseq analysis of KEGG differential metabolic pathways, comparison between SHL medium-dose groups and inflammation model groups.

In order to explore whether the antipyretic and anti-inflammatory effects of SHL are affected by the gut microbiota, the 16S rRNA gene sequence of fecal samples of rats in different groups was analyzed. At the phylum level, the global community structure of the sample showed that the proportion of *Bacteroides* and *Firmicutes* accounted for about 90%. Compared with the CG, the ratio of *Firmicutes* to *Bacteroides* in the MG increased, and the ratio of *Proteobacteria* to *Actinobacteria* decreased. After the treatment of SHL, the ratios all returned to the level of the CG, maintaining the steady state of the microbiota (Figure 5A). In addition, the alpha diversity of the samples was assessed at the OTU level. The index refers to the diversity within habitat or intra-community, and it is in direct proportion to species richness. The results showed that there had a high degree of microbial community richness, and the Kruskal–Wallis test showed no significant difference in community richness among groups (Supplementary Figure S5). Due to the low OTU abundance in the C1 sample and the large difference in the blank group, C1 was eliminated in subsequent analysis.

Principal coordinate analysis (PCOA) based on the unweighted UniFrac distance exhibited that there was a significant difference between CG and MG. After SHL treatment, the gut microflora structure of the MG was significantly changed. Principal components 1 (PC1) and PC2 accounted for 16.9 and 14.1% of the total variance respectively, explaining a total of 30% of the variation (Figure 5B).

Biomarker discovery associated with inflammation was conducted by linear discriminant analysis (LDA) effect size (LEfSe), which was used to determine the significant differences among different groups. As shown in Figure 5C and Supplementary Figure S6, compared with the CG, the diversity of community composition in the MG was mainly as follows: at the genus level, the abundance of *Bifidobacterium*, *Olsenella*, *Lacticigenium*, and *Isobaculum* in the MG was significantly higher than that in the CG (*p < 0.05) and the abundance of *Bacteroides*, *Subdoligranulum,* and *Faecalibacterium* decreased significantly (*p < 0.05). *Bifidobacterium*, *Olsenella*, *Bacteroides*, the unknown *P_75_a5* species, *Clostridium*, *Enterococcus*, and *Aerococcus* levels were restored by treating with SHL. Meanwhile, the abundance of probiotics *Roseburia* was significantly increased (*p < 0.05), the pathogenic abundance of *Turicibacter*, *Allobaculum*, *Jeotgalicoccus*, *Lactococcus*, the unknown *P_75_a5* species, *Clostridium* and *Ralstonia* decreased significantly (*p < 0.05) (Figure 5D, Supplementary Figure S7). In general, SHL can regulate the gut microbiota dysbiosis of LPS-induced inflammatory injury in rats.

These microbiota were ranked based on their variable importance in genus-level as follows: *Olsenella* > *Clostridium* > *Enterococcus* > *Bifidobacterium* > *Aerococcus*. MetagenomeSeq method was used to analyze the differential metabolic pathways among each group. According to the criteria of *p < 0.05, the differential metabolic pathways in the KEGG and MetaCyc pathways between the MG and the ZG were mainly reflected as follows: the Geraniol degradation pathway was significantly upregulated, and the proteasome and naphthalene degradation pathways were significantly downregulated in the ZG (Figure 5E).

### Relevance Analysis Between Serum Biomarkers and Pro-inflammatory Cytokines

Taking the content of cytokines IL-6, TNF-α, and IL-1β as the X variable, and the peak area of the different metabolites in each group as the Y variable, the data matrix was performed to z-score standardization for further analysis. Spearman correlation analysis was used to calculate the XY data matrix correlation and *p*-value, and the specific results are shown in Supplementary Table S2. N-acetylserotonin was significantly negatively correlated with IL-6 and IL-1β (*p < 0.05). 4-hydroxycinnamic acid was significantly negatively correlated with IL-6, TNF-α, and IL-1β (**p < 0.01, ***p < 0.001, and *p < 0.05). Uric acid was significantly positively correlated with IL-6 and TNF-α (*p < 0.05). N-acetylserotonin and 4-hydroxycinnamic acid showed a significant negative correlation with all pro-inflammatory cytokines. These two endogenous metabolites are significantly decreased in the MG (compared to the CG), resulting in increased pro-inflammatory cytokines and heat transfer in MG. In addition, 1-methylxanthine and uric acid had a significant positive correlation with IL-6 and TNF-α (*p < 0.05, **p < 0.01). However, the levels of TNF-α were significantly positively correlated with cytidine (**p < 0.01). Therefore, it is speculated that 4-hydroxycinnamic acid, uric acid, N-acetylserotonin, 1-methylxanthine, and cytidine are biomarkers exhibiting antipyretic and anti-inflammatory therapeutic effects of SHL. In general, the current analysis found strong evidence of a correlation between serum biomarkers and pro-inflammatory cytokines.

### Relevance Analysis Between Serum Biomarkers and Gut Microbiota

In order to comprehensively analyze the relationship between eight biomarkers and 12 kinds of gut microbiota, a correlation matrix was established by calculating the Spearman correlation coefficient, as shown in Supplementary Table S3. The results showed that pyrrolidone carboxylic acid was significantly positively correlated with *Actinobacteria*, *Bifidobacteriumles*, *Bifidobacteriumceae*, *Enterococcaceae*, and *Bifidobacterium* (*p < 0.05), and significantly negatively correlated with *Bacteroidaceae* and *Bacteroides* (*p < 0.05). N-acetylserotonin was significantly negatively correlated with *Actinobacteria*, *Bifidobacteriumles*, *Bifidobacteriumceae*, *Enterococcaceae*, *Bifidobacterium*, *Olsenella*, *Enterococcus* (*p < 0.05). Cytidine and *Olsenella* presented significant negative correlations (*p < 0.05). 1-methylxanthine was significantly positively correlated with *Bifidobacteriumles*, *Bifidobacteriumceae*, and *Bifidobacterium* (*p < 0.05) and significantly negatively correlated with *Bacteroidaceae*, *Olsenella*, *Bacteroides*, and *Clostridium* (*p < 0.05). Observations such as these underscored the close association between the metabolites and gut microbiota.

## Discussion

In recent years, a large number of studies have demonstrated that the occurrence and development of inflammation are closely associated with the gut microbiota (Dinh et al., 2015; Shin et al., 2015; Tilg et al., 2020). Some studies have confirmed that the response of the gut microbiota to all inflammation models appears to be similar, indicating that the underlying mechanism may be non-specific (Lupp et al., 2007). Gut microbiota may generate a vital role in the prevention and treatment of inflammation by acting on the endocrine system, nervous system and immune pathways (Richards et al., 2016; Pickard et al., 2017). This study is the first to reveal the antipyretic and anti-inflammatory mechanisms of SHL from the perspectives of gut microbiota and serum metabolites.

In our experiments, it was confirmed that SHL could reduce the pro-inflammatory cytokines TNF-α, IL-6, and IL-1β in LPS-induced fever rats in order to play an antipyretic and anti-inflammatory effect. Subsequently, we identified 39 different metabolites related to inflammation in rat serum, and SHL had a therapeutic effect on the changes of eight metabolites induced by LPS. Spearman analysis indicated that five metabolites (4-hydroxycinnamic acid, uric acid, N-acetylserotonin, 1-methylxanthine, and cytidine) were significantly correlated with three pro-inflammatory cytokines, finding that these affected biomarkers were mainly involved in the pathway of riboflavin metabolism, D-glutamine and D-glutamate metabolism, caffeine metabolism, ABC transporters, and vitamin rare earths and absorption.

Our findings have been preliminarily confirmed in other studies. It has been reported that riboflavin also exhibits activity in the systemic inflammation models and reduces the LPS-induced synthesis of pro-inflammatory cytokines TNF-α, IL-1, and IL-6 (Toyosawa et al., 2004a; Toyosawa et al., 2004b; Kodama et al., 2005). As discovered by Moïse et al. (Co ë ffier et al., 2001)*,* glutamine restricted the secretion of pro-inflammatory cytokines IL-6 and IL-8 by intestinal cells *via* the post-transcriptional pathway, which might be applied to regulate imbalanced inflammatory conditions caused by cytokines. Hydroxycinnamic acid derivatives downregulated the expression of TNFα, monocyte chemoattractant protein-1 (MCP-1), and plasminogen activation inhibitor-1 (PAI-1), and increased the production of the anti-inflammatory adiponectin in fat cells. Liu et al. (2021) found that N-acetylserotonin could reduce the expression of IL-1β through the TLR4/NF-κB/NLRP3 pathway, which was consistent with the results of this study. In addition, the conversion of 1-methylxanthine to 1-methyluric acid by xanthine oxidase led to endothelial damage directly and/or by triggering the accumulation of inflammatory cells (Németh and Boda, 2001). The elevated uric acid in the acute phase caused intestinal epithelial cell inflammation and subsequently the release of IL-1β. The mechanism of this reaction was to increase the production of mitochondrial ROS by upregulating the expression of TSPO and to activate the NF-kB pathway (Chen et al., 2018).

Then, we used 16s rRNA sequencing to record the changes in the structure and function of the gut microbiota in the inflammatory hyperthermia rat model. At the phylum level, the abundance of *Actinobacteria* increased significantly in the model group compared with the blank group, and the *Firmicutes* and *Bacteroidetes* decreased significantly in comparison with the SHL treatment group. Disorders of the host gut microbiota caused by inflammatory hyperthermia might encourage the proliferation of other low-abundance and pathogenic bacteria (such as *Olsenella*), thereby further exacerbating the inflammatory response. SHL could significantly enhance the systemic inflammatory response caused by LPS by adjusting the composition, abundance, and abnormality of gut microbiota. As revealed by the relationship between previous endogenous metabolites and pro-inflammatory cytokines, N-acetylserotonin was significantly negatively correlated with IL-6 and IL-1β (*p < 0.05). In addition, 1-methylxanthine was significantly positively correlated with IL-6 and TNF-α (*p < 0.05, **p < 0.01). Correlation analysis was also conducted to analyze the relationships between endogenous metabolites, gut microbiota, and pro-inflammatory cytokines. The possible antipyretic and anti-inflammatory mechanisms of SHL were stated as follows. SHL can downregulate the abundances of *Bifidobacterium*, *Olsenella*, and *Enterococcus* in LPS-stimulated rats with inflammation and can upregulate that of Bacteroides, thereby affecting the levels of endogenous metabolites N-acetylserotonin and 1-methylxanthine involved in the tryptophan metabolism and caffeine metabolism pathways. In this way, pro-inflammatory cytokines can be inhibited.

In this study, it was confirmed the increase of intestinal *Bifidobacterium* in the MG might be caused by the performance of the barrier protection function of the intestinal mucosa in the anti-inflammatory effect after the occurrence of intestinal inflammation. Biagi et al. (2010) suggested that the Toll-like receptor TLR4 could recognize lipoteichoic acid and peptide polysaccharides, which were mainly derived from gram-negative bacterial cell wall components, such as LPS. It was speculated that the increased abundance of *Bifidobacterium* in MG might be related to the expression of Toll-like receptors (TLRs). After treatment with SHL, the abundance of *Bifidobacterium* decreased, which might be associated with its bitter flavor and cold property. Studies have reported that Chinese botanical drugs with a bitter flavor and cold property have inhibitory effects on the probiotics in the intestines. For example, the long-term application of Huanglian Jiedu Decoction reduces the number of intestinal probiotics such as *Lactobacillus* and *Bifidobacterium* (Luo et al., 2009; Xie et al., 2011). The downregulation of *Olsenella*, a conditional pathogen, can reduce the possibility of inflammation in the rumen epithelium and the organism, which is linked with the inhibition of bacterial virulence genes (Mocanu et al., 2021). *Enterococcus* can suppress the production of pro-inflammatory cytokine TNF-α, leading to increased inflammation and decreased intestinal integrity (Marcinkiewicz et al., 2007). In a word, it is a more feasible strategy for SHL to ameliorate inflammatory disease and related hyperpyrexia response by regulating gut microbial distribution exerted by probiotics.

This study aimed to evaluate the specific interaction between LPS-induced inflammatory hyperthermia and gut microbiota based on 16S rRNA gene sequencing combined with UHPLC-MS-based metabolomics and to explore the internal regulatory mechanism of SHL in the inflammation-disturbed gut microbiota. As a result, it was found that rats with inflammation exhibited obvious gut microbiota disorder and abnormal intestinal metabolic profile. The antipyretic and anti-inflammatory effects of SHL might be related to its regulation of the alpha and beta diversities of the microbiota, which reduced the abundance of pathogenic bacteria (*Olsenella* and *Enterococcus*) and increased those of probiotics (*Bacteroides*). In turn, it affected the recovery of the abnormal levels of endogenous metabolites (N-acetylserotonin and 1-methylxanthine) in the tryptophan metabolism and caffeine metabolism pathways, thus inhibiting the pro-inflammatory cytokines. Moreover, this work provides new evidence for the study of the mechanism of the therapeutic effect of SHL or other botanical drugs with low bioavailability. In future studies, we will conduct microbial transplantation experiments to confirm the roles of characteristic intestinal microflora in the treatment of inflammation, which are expected to provide theoretical support for the application of microbial therapy in inflammatory diseases.

## Data Availability

The original contributions presented in the study are included in the article/Supplementary Material; further inquiries can be directed to the corresponding author.
