# Supplementary material for: Study on the Antipyretic and Anti-inflammatory Mechanism of Shuanghuanglian Oral Liquid Based on Gut Microbiota-Host Metabolism
Source: Front Pharmacol. 2022 Jun 28;13:843877. doi: 10.3389/fphar.2022.843877 (PMC9273999; doi:10.3389/fphar.2022.843877)
Supplement: Supplementary file 1 [file DataSheet1.pdf]

Study on the Antipyretic and Anti-inflammatory Mechanism of Shuanghuanglian Oral Liquid Based on Gut Microbiota -Host Metabolism

Yan Gao [1], *Lu Liu* [1#], Jing Lv[1], *Long-fei Yang* [1], *Chen Li*[2], *Yu-ting Liang*[1], and *Bo-nian Zhao*[1]*

[1] Shandong University of Traditional Chinese Medicine, Jinan 250355, China;

[2] Shandong Mingren Freda Pharmaceutical Co, Ltd, Jinan, Shandong, China.

***Correspondence**: bonianzh@163.com

[#]These authors contributed to the work equality and should be regarded as co-first authors.

Keywords: Shuanghuanglian oral liquid, Metabolomics, Gut microbiota, Antipyretic, Anti-inflammatory

Figure legends

Figure S1. The levels of pro-inflammatory cytokines of serum samples at different points for blood sampling. (A: IL-1β; B: IL-6; C: TNF-α). Data was expressed as mean ± SD (*P<0.05 model group compared with the SHL medium dose group, CG: normal control group, MG: model group, ZG: SHL medium dose group).

Figure S2. The base peak intensity (BPI) chromatograms of serum samples from each groups. A: in positive mode; B: in negative mode

Figure S3. Metabolic profiles of serum samples in rats with inflammation induced by lipopolysaccharide. A: PCA score plot in positive ion mode; B: PCA score plot in negative ion mode.

Figure S4. Permutation test results of the OPLS-DA model. A: Permutation test results between control groups and model groups in positive mode; B: Permutation test results between control groups and model groups in negative mode; C: Permutation test results between model groups and SHL medium dose groups in positive mode; D: Permutation test results between model groups and SHL medium dose groups in negative mode

Figure S5. Box plot of alpha diversity index of bacteria

Figure S6. Specific levels of differential gut microbiota between the normal control groups and model groups. Data was expressed as mean ± SD (#P<0.05 between the normal control groups and model groups). A: Subdoligranulum; B: Bacteroides; C: Bacteroidaceae; D: Olsenella; E: Isobaculum; F: YS2; G: Cyanobacteria; H: Actinobacteria; I: Faecalibacterium; J: Lachnospiraceae; K: Bifidobacteriales; L: Bifidobacteriales; M: Lacticigenium; N: Bifidobacteriaceae; O: Bifidobacterium; P: Actinobacteria.

Figure S7. Regulation of SHL on intestinal bacteria in the model group. Data was expressed as mean ± SD ($^{*}P$<0.05 between the model groups and Shuanghuanglian treatment groups). A: Bacillaceae; B: Bifidobacteriaceae; C: Enterococcaceae; D: Bacteroidaceae; E: Actinobacteria; F: Bifidobacteriales; G: Bifidobacterium; H: Enterococcus; I: Aerococcus; J: Olsenella; K: Clostridium; L: Bacteroides

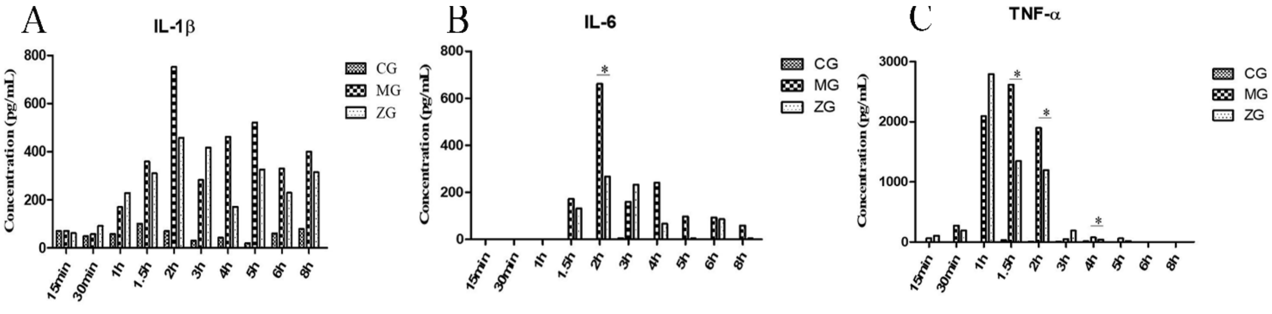

Figure S1. The levels of pro-inflammatory cytokines of serum samples at different points for blood sampling. (A: IL-1β; B: IL-6; C: TNF-α). Data was expressed as mean ± SD (*P<0.05 model group compared with the SHL medium dose group, CG: normal control group, MG: model group, ZG: SHL medium dose group).

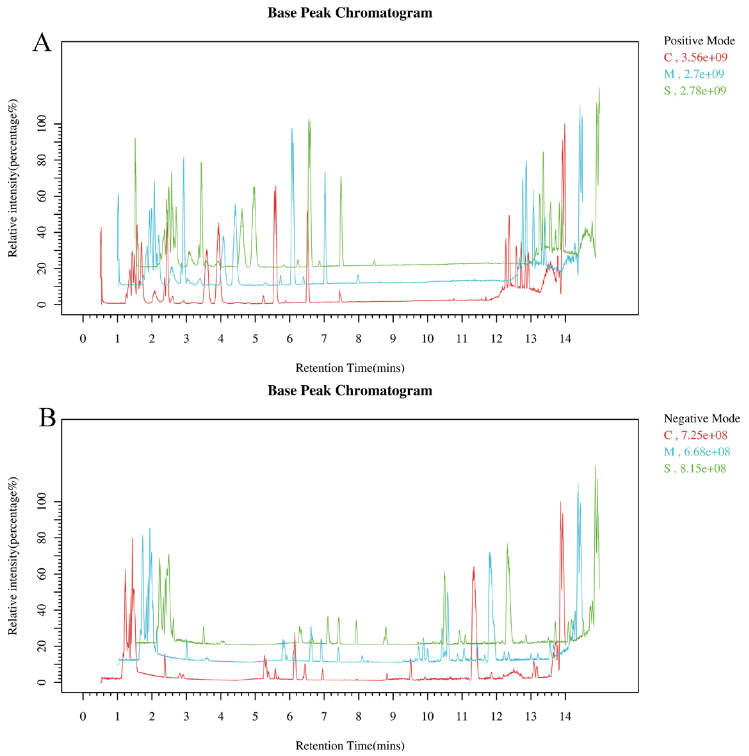

Figure S2. The base peak intensity (BPI) chromatograms of serum samples from each groups. A: in positive mode; B: in negative mode

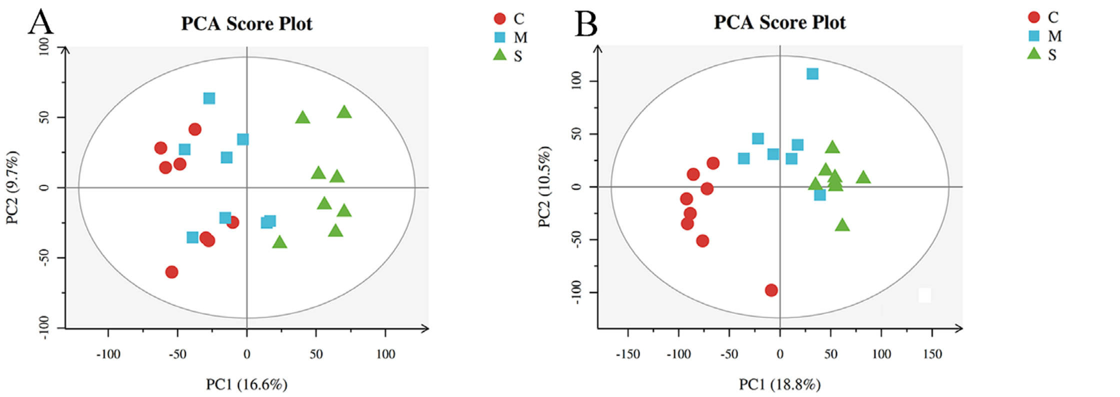

Figure S3. Metabolic profiles of serum samples in rats with inflammation induced by lipopolysaccharide. A: PCA score plot in positive ion mode; B: PCA score plot in negative ion mode.

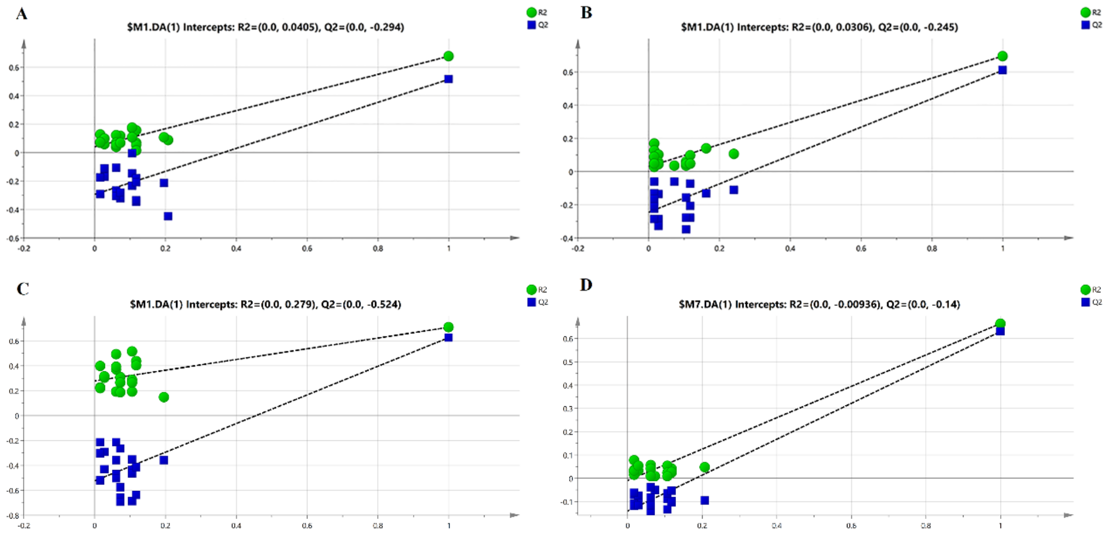

Figure S4. Permutation test results of the OPLS-DA model. A: Permutation test results between control groups and model groups in positive mode; B: Permutation test results between control groups and model groups in negative mode; C: Permutation test results between model groups and shuanghuanglian treatment groups in positive mode; D: Permutation test results between model groups and shuanghuanglian treatment groups in negative mode

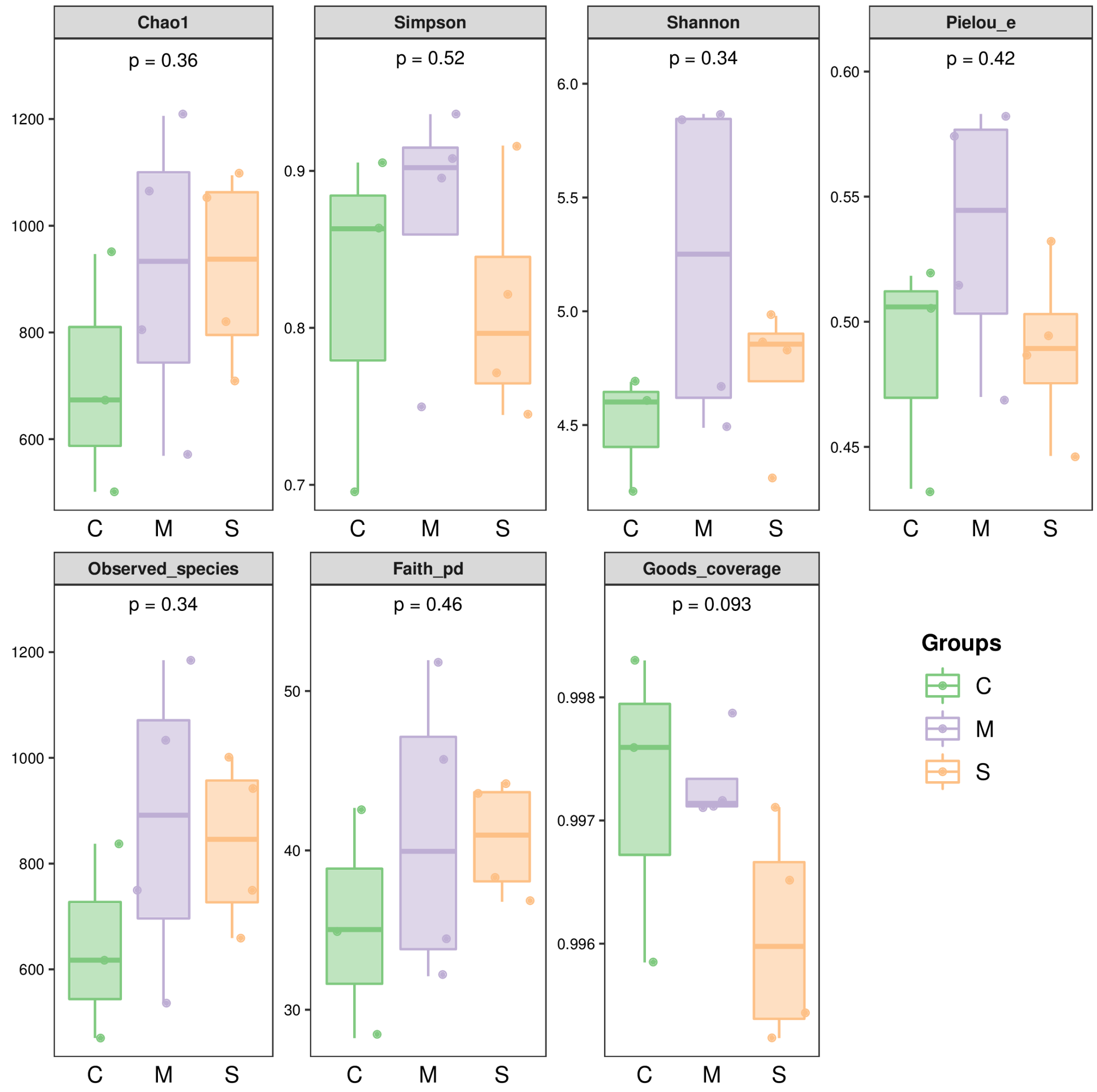

Figure S5. Box plot of alpha diversity index of bacteria

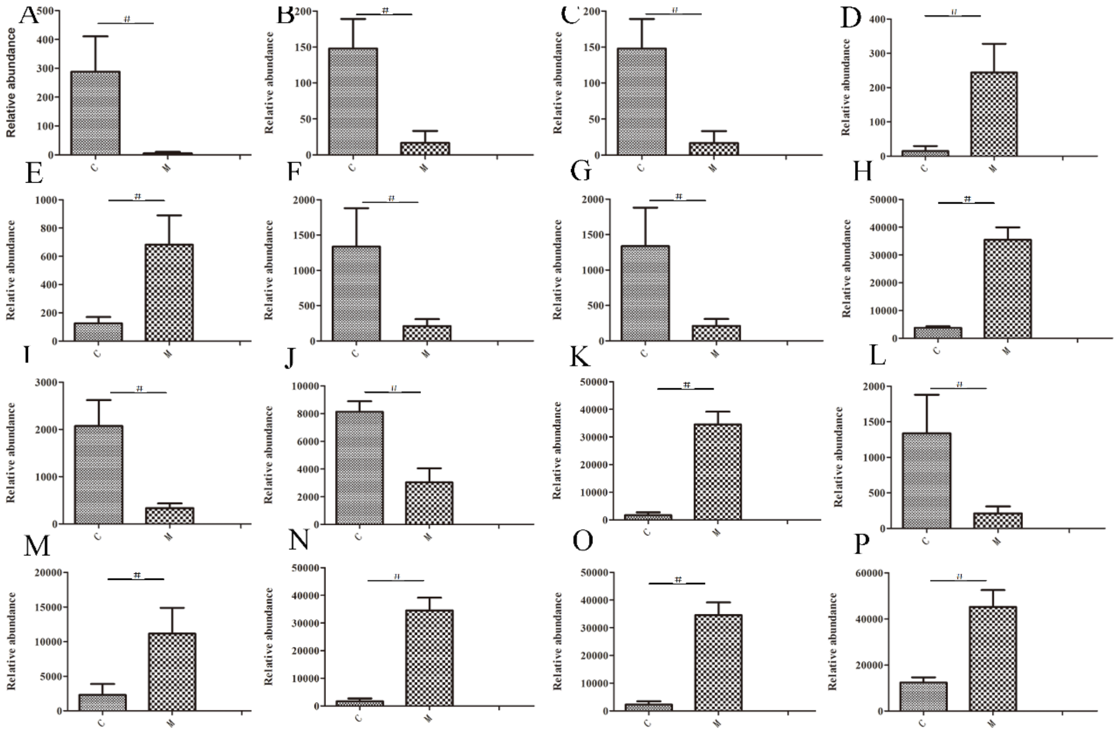

Figure S6. Specific levels of differential gut microbiota between the normal control groups and model groups. Data was expressed as mean ± SD ($^{\#}P$<0.05 between the normal control groups and model groups). A: Subdoligranulum; B: Bacteroides; C: Bacteroidaceae; D: Olsenella; E: Isobaculum; F: YS2; G: Cyanobacteria; H: Actinobacteria; I: Faecalibacterium; J: Lachnospiraceae; K: Bifidobacteriales; L: Bifidobacteriales; M: Lacticigenium; N: Bifidobacteriaceae; O: Bifidobacterium; P: Actinobacteria.

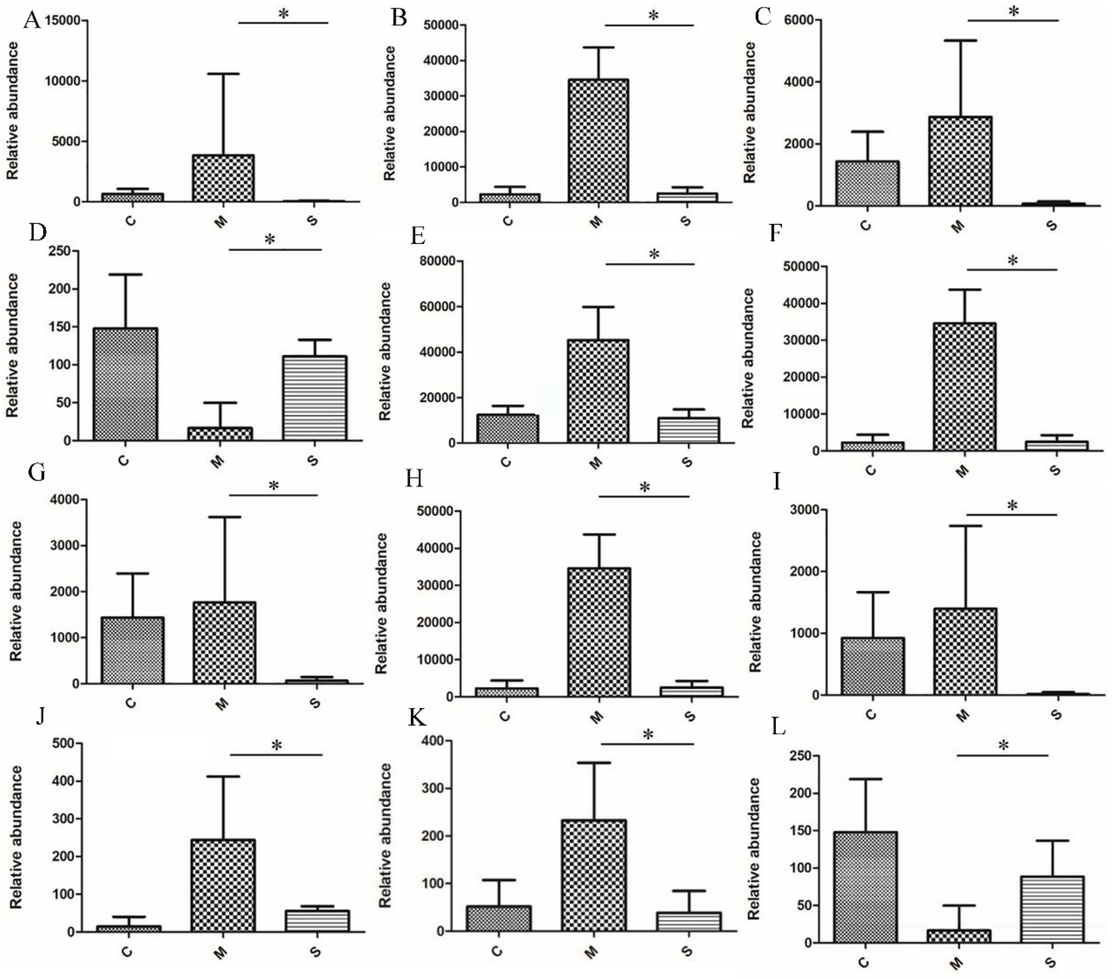

Figure S7. Regulation of SHL on intestinal bacteria in the model group. Data was expressed as mean ± SD ($^{*}P$<0.05 between the model groups and Shuanghuanglian treatment groups). A: Bacillaceae; B: Bifidobacteriaceae; C: Enterococcaceae; D: Bacteroidaceae; E: Actinobacteria; F: Bifidobacteriales; G: Bifidobacterium; H: Enterococcus; I: Aerococcus; J: Olsenella; K: Clostridium; L: Bacteroides

Table legends

Table S1. The temperature difference to time (ΔT) of rats in each groups (n = 8)

Table S2. Correlation analysis between characteristic metabolites and pro-inflammatory cytokines

Table S3. Correlation analysis between characteristic metabolites and gut microbiota

Table S1. The temperature difference to time (ΔT) of rats in each groups (n = 8)

No.	0.5h	1h	1.5h	2h	2.5h	3h	3.5h	4h	4.5h	5h	5.5h	6h	6.5h	7h	7.5h	8h
**CG**	**0.22±0.72**	**0.36±0.79**	**-0.06±0.82**	**0.24±0.54**	**0.24±0.95**	**0.16±0.67**	**0.24±0.86**	**0.26±0.89**	**-0.06±0.87**	**-0.06±0.65**	**0.10±0.78**	**0.04±0.82**	**0.44±1.05**	**0.50±0.89**	**0.56±1.00**	**0.28±0.84**
**MG**	**0.57±0.70**	**0.85±0.71**	**0.63±0.99**	**1.18±1.42**	**1.03±0.89**	**0.60±0.78**	**0.35±0.88**	**0.75±0.88**	**1.52±0.74**	**1.48±0.81**	**1.78±0.74**	**1.70±0.86**	**1.00±0.66**	**1.27±0.60**	**1.08±0.69**	**1.07±0.65**
**PG**	**0.45±0.32**	**0.20±0.55**	**0.43±0.77**	**0.98±0.71**	**0.62±0.74**	**0.17±1.05**	**0.00±1.00**	**-0.02±0.64**	**0.65±0.42**	**0.65±0.52**	**0.80±0.65**	**0.97±0.53**	**0.63±0.39**	**0.47±0.48**	**0.67±0.54**	**0.62±0.59**
**LG**	**0.98±0.64**	**0.77±0.49**	**1.00±0.69**	**1.23±0.95**	**1.50±1.04**	**0.98±1.07**	**0.97±0.99**	**0.90±0.77**	**1.43±0.85**	**1.48±0.80**	**1.67±1.06**	**1.80±0.99**	**1.53±0.77**	**1.33±0.78**	**1.57±0.75**	**1.43±0.52**
**ZG**	**0.35±0.55**	**0.2±0.88**	**0.15±1.63**	**0.65±1.25**	**0.37±1.19**	**0.42±0.88**	**0.42±0.82**	**0.37±0.68**	**0.60±0.69**	**0.90±0.70**	**1.07±0.73**	**1.17±0.78**	**0.92±0.88**	**0.80±0.77**	**0.88±0.81**	**0.85±0.74**

Table S2 Correlation analysis between characteristic metabolites and pro-inflammatory cytokines

Name	IL-6		TNF-α		IL-1β	
	r	*P*	r	*P*	r	*P*
Uric acid	0.4914	0.0147*	0.4922	0.0146*	0.2148	0.3134
Riboflavin	0.3349	0.1097	0.2487	0.2413	0.1957	0.3594
Pyrrolidonecarboxylic acid	0.1831	0.3918	0.2991	0.1556	0.2022	0.3433
N-Acetylserotonin	-0.4153	0.0436*	-0.3122	0.1375	-0.4610	0.0234*
Cytidine	0.3918	0.0583	0.5757	0.0032**	0.1661	0.4379
4-Hydroxycinnamic acid	-0.6188	0.0013**	-0.7087	0.0001***	-0.4975	0.0134*
1-Methylxanthine	0.4836	0.0167*	0.6148	0.0014**	0.1026	0.6332
Palmitoleic acid	0.3334	0.1114	0.353	0.0906	0.0795	0.7118

Note: r: correlation coefficient; *P*: Correlation coefficient significance; **P*<0.05, ***P*<0.01, ****P*<0.001

Table S3 Correlation analysis between characteristic metabolites and gut microbiota

Name	Uric acid		Riboflavin		Pyrrolidonecarboxylic acid		N-Acetylserotonin		Cytidine		4-Hydroxycinnamic acid		1-Methylxanthine		Palmitoleic acid	
	r	*P*	r	*P*	r	*P*	r	*P*	r	*P*	r	*P*	r	*P*	r	*P*
P.actinobacteria	0.3548	0.2843	0.4803	0.1348	0.6669	0.0250*	-0.6726	0.0233*	0.4495	0.1655	-0.241	0.4753	0.5964	0.0528	0.3704	0.2621
O.bifidobacteriales	0.3638	0.2715	0.5108	0.1083	0.6818	0.0209*	-0.6166	0.0433*	0.5706	0.0668	-0.2239	0.5081	0.6655	0.0254*	0.4423	0.1732
F.bifidobacteriaceae	0.3638	0.2715	0.5108	0.1083	0.6818	0.0209*	-0.6166	0.0433	0.5706	0.0668	-0.2239	0.5081	0.6655	0.0254*	0.4423	0.1732
F.bacteroidaceae	0.3638	0.2715	0.5108	0.1083	0.6818	0.0209*	-0.6166	0.0433	0.5706	0.0668	-0.2239	0.5081	0.6655	0.0254*	0.4423	0.1732
F.bacillaceae	0.1103	0.7469	0.3535	0.2862	0.5033	0.1145	-0.5193	0.1017	0.242	0.4735	0.0625	0.8551	0.0727	0.8318	-0.0818	0.8111
F.enterococcaceae	-0.1289	0.7056	0.3799	0.2491	0.6049	0.0487*	-0.7394	0.0093**	-0.0082	0.9809	0.2599	0.4401	0.0444	0.8969	-0.1062	0.756
Bifidobacterium	0.3638	0.2714	0.5109	0.1083	0.6817	0.0209*	-0.6165	0.0434*	0.5705	0.0669	-0.2238	0.5082	0.6655	0.0254*	0.4423	0.1731
Olsenella	-0.2866	0.1177	0.0192	0.1681	0.1795	0.0622	-0.0264	0.0220*	-0.4926	0.0136*	0.18	0.1374	-0.2029	0.0014**	-0.3079	0.1004
Bacteroides	-0.2995	0.3709	-0.3782	0.2514	-0.7482	0.0081*	0.531	0.0928	-0.2097	0.536	0.1373	0.6874	-0.628	0.0385*	-0.5108	0.1083
Aerococcus	-0.1823	0.5916	0.3394	0.3072	0.4975	0.1195	-0.5101	0.1089	0.1529	0.6537	0.2033	0.5488	-0.0071	0.9836	-0.3297	0.3221
Enterococcus	-0.2817	0.4013	0.1846	0.5868	0.5497	0.0798	-0.6083	0.0470*	-0.1304	0.7023	0.2945	0.3794	-0.1232	0.7182	-0.2134	0.5287
Clostridium	-0.408	0.2129	-0.2514	0.4559	-0.0406	0.9058	-0.0297	0.931	-0.5005	0.1169	0.2276	0.501	-0.6176	0.0429*	-0.5213	0.1001

Note: r: correlation coefficient; *P*: Correlation coefficient significance; *P<0.05, **P<0.01, ***P<0.001
